# Bi-Directional Loop Antenna Array Using Magic Cube Origami

**DOI:** 10.3390/s19183911

**Published:** 2019-09-11

**Authors:** Ying Xu, Yeonju Kim, Manos M. Tentzeris, Sungjoon Lim

**Affiliations:** 1 School of Electrical and Electronics Engineering, College of Engineering, Chung-Ang University, Seoul 06974, Korea; 2 School of Electrical and Computer Engineering, College of Engineering, Georgia Institute of Technology, Atlanta, GA 30332, USA

**Keywords:** bidirectional antenna array, origami antenna, loop antenna, magic cube

## Abstract

In this paper, we propose a bi-directional loop antenna array using magic cube origami. The proposed antenna array consists of three one-wavelength loop antenna elements with series feeding. Each loop antenna is realized on a single magic cube, and three cubes are connected in series to form the array. The three cubes can be easily folded and unfolded due to being constructed in the form of a magic cube origami. Antenna volume can be minimized for high mobility by folding the array, which radiates a bi-directional pattern with full volume when unfolded. The proposed antenna was designed at 1.39 GHz. When the single antenna is realized on the single cube, the peak gain is 4.03 dBi. The peak gain increased to 5.2 and 5.53 dBi with two and three antennas, respectively. Half-power beam width (HPBW) with three antenna elements decreased to 40° from 360° compared to the HPBW with the single antenna. The proposed antenna performance was assessed numerically and experimentally.

## 1. Introduction

Different antenna forms are required to achieve signal coverage for special environments overall communication quality [1,2]. Antennas with bi-directional radiation are useful for many applications, including wireless communication [3,4], microwave sensor networks [5], and broadcasting base stations [6,7]. For example, in reference [8], the antennas realized circular polarized (CP) radiation of the same sense by using techniques of exciting two back-to-back perturbed patch antennas with single-layer or multilayered substrates. However, generally, such antenna structures cannot be folded and unfolded using origami principles.

Origami is an ancient oriental art of folding paper to create various three-dimensional (3D) shapes, and it has recently been applied in mathematics, machinery, mechanics, materials, control, biology, medicine, and other basic intersection fields. For example, Ryan et al. used Mura origami structure to achieve acoustic field morphing [9]; Felton et al. utilized origami technology to create self-folding devices [10]. Hanna et al. proposed an origami waterbomb base (i.e., a single-vertex bi-stable origami mechanism) with unique properties, which are promising for various applications [11].

Electrical engineers are also actively seeking ways to apply origami structures for antennas, sensors, and absorbers [12,13,14]. For instance, a dual-band reconfigurable origami antenna with changeable resonant frequency has been proposed by changing the folding states of a magic cube [15]. Liu et al. introduced a reconfigurable multi-radii helical origami antenna by varying the helical origami height [16]. A dual–mode reconfigurable origami antenna provides directional mode(s) when the antenna is folded and omnidirectional when totally unfolded, with additional switching frequency ability; a dual–mode reconfigurable origami antenna [17], providing directional mode(s) when the antenna is folded and omnidirectional when totally unfolded as well as switching operational frequencies. In addition, a mode-reconfigurable origami spiral antenna has been proposed [18]. From the developed (unfolded) state to the collapsed (folded) state, the realized gain is from 6.2 to 8.9 dBi, meanwhile its main beam is reconfigurable from two to one.

This paper proposes a bi-directional loop antenna array using magic cube origami. The novelty of the proposed deployable origami antenna is the series-fed array configuration. To realize the deployable series-fed array configuration, we introduced the magic cube origami. Each loop antenna is realized on the single magic cube, and three magic cubes are connected in the series. The loop type antenna is employed on the magic cube for a series array, while a monopole type antenna is employed on the magic cube in reference [15] for frequency reconfigurability. Based on the configuration of the magic cube origami, it can be easily folded and unfolded, minimizing overall volume in the folded state for high mobility, and radiating a bi-directional pattern with full volume when unfolded. Therefore, the proposed antenna is useful for deployable antenna applications and shows high mobility. For instance, it can be folded to minimize the overall volume when it is carried and then unfolded in use to maximize the radiation performance. In the unfolded state, the proposed antenna operates at 1.39 GHz, where some potential applications can be found, for instance, retinal prosthesis [19]. The performance of the proposed antenna is demonstrated numerically and experimentally in terms of peak gain and the half-power beam width (HPBW).

## 2. Antenna Array Design

**Figure 1:**
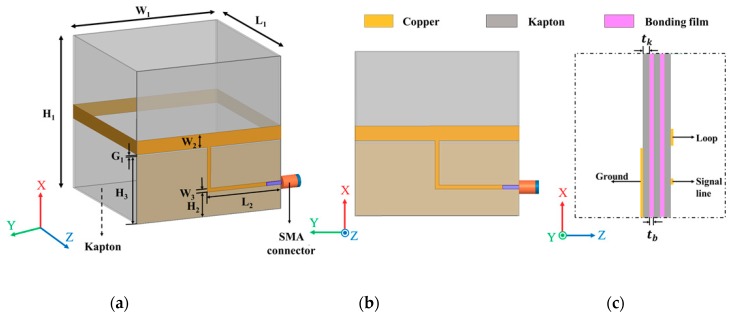
Illustration of the single magic cube antenna: (**a**) Angled view, (**b**) front view, and (**c**) overlapping layers at z = L_1_ and y = W_1._

The presented antenna was designed on a Kapton substrate in an origami magic cube shape, which had six square-shaped sides. Meanwhile, the dimensions of all sides were the same. The one-wavelength loop antenna was realized on the magic cube. Because the total length of the loop antenna was quadruple of the magic cube size (L_1_), the resonant frequency could be designed by the size of the magic cube. Therefore, W_1_ was chosen as 50 mm to resonate at 1.39 GHz. According to the principle of pattern multiplication, the radiation pattern of an array is the product of the loop antenna element pattern and array factor pattern. Therefore, the array factor was calculated from the loop antenna element pattern for higher directivity and narrower beam width. In this work, the phase difference between each loop antenna element was 180°. Next, the feeding lines were designed on the magic cubes to realize phase difference. Because of the magic cube geometry, the feeling line design must be limited. Finally, the antenna array with the feeding lines were designed and optimized to achieve impedance matching. Figure 1 illustrates the single origami antenna geometry in the unfolded state.

As shown in Figure 1a, the antenna is fed by a microstrip line realized on a Kapton film. Since the magic cube width (W_1_) = length (L_1_), total loop length = 4 W_1_, and loop width = W_2_. Figure 1b shows the front view, where the microstrip line was realized with a sub-miniatured version A (SMA) connector. As shown in Figure 1c, three Kapton films were bonded, and the overall substrate thickness was 0.52 mm because the thickness of Kapton (t_k_) and bonding films (t_b_) were 0.12 mm and 0.08 mm, respectively. The width (W_3_) of the microstrip line was designed to have 50 Ω characteristic impedance. If the thickness was too thin, it was difficult to fabricate a 50 Ω microstrip line because of a narrow W_3_. Therefore, we set W_3_ to be 1.2 mm with the overall substrate thickness of 0.52 mm.

In this work, ANSYS high frequency structure simulation (HFSS) software was used to design and analyze the proposed antenna, with dielectric constant and tangential loss of the Kapton film set of *ε*_r_ = 3.5 and *tan δ* = 0.002, respectively, and *ε*_r_ = 3 and *tan δ* = 0.05, respectively, for the double-sided bonding film. The conductive pattern was realized by 0.1 mm thick copper film with 5.8 × 10^7^ S/m conductivity.

**Figure 2:**
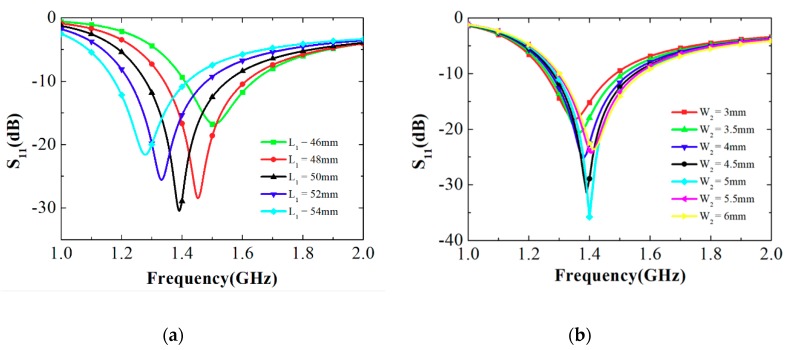
Simulated S-parameters of the proposed loop antenna at different parameters of (**a**) L_1_ and (**b**) W_2_ in the unfolded state.

Figure 2 shows the simulated S-parameters of the proposed loop antenna at different parameters of L_1_ and W_2_ in the unfolded state. It is observed from Figure 2a that the resonant frequency decreased as L_1_ increased. Compared to W_2_, L_1_ had a more dominant influence on the resonant frequency. In the study, L_1_ was chosen as 50 mm to get the designed operation frequency of 1.39 GHz, with the value of W_2_ being 4.5 mm. In addition, the parametric study for W_2_ had been simulated with the value of L_1_ is 50mm. It is observed from Figure 2b that W_2_ affected the impedance of the proposed antenna. In this work, W_2_ = 4.5 mm to achieve good impedance matching and a wider impedance bandwidth.

**Figure 3:**
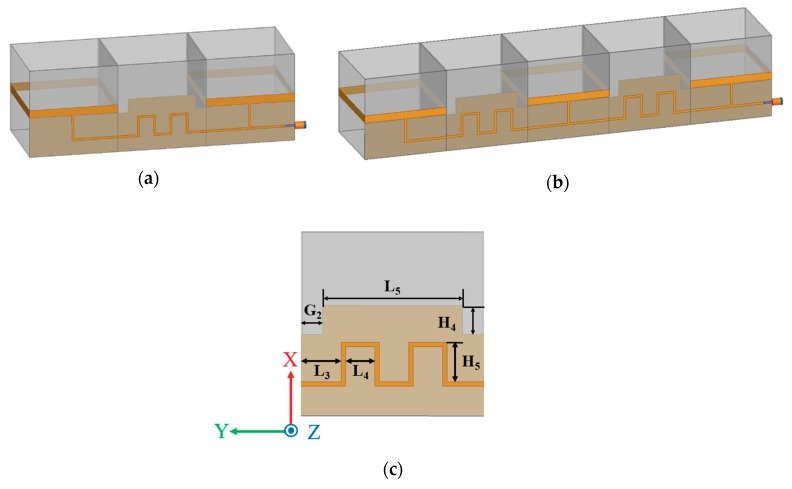
Illustration of the (**a**) two-element antenna array and (**b**) three-element antenna array. (**c**) Layout of the series feeding line.

Figure 3a,b show illustrations of the two-element antenna array and three-element antenna array, respectively, and Figure 3c shows the layout of the series feeding line. The geometrical parameters of the single antenna in Figure 1 are W_1_ = H_1_ = L_1_ = 50 mm, W_2_ = 4.5 mm, W_3_ = 1.2 mm, H_2_ = 8 mm, H_3_ = 22.15 mm, L_2_ = 25.6 mm, and G_1_ = 0.6 mm. The radiation pattern of the single antenna was omnidirectional, a bi-directional radiation pattern was obtained with the microstrip fed loop antenna configuration for the two-elements and three-elements antenna array. The two-element antenna array was realized by connecting three identical origami magic cubes as shown in Figure 3a. Figure 3b shows the series feeding line to achieve a maximum peak gain of the proposed antenna array. Figure 3c shows the interconnect line between magic cube antennas. For impedance matching, its geometric parameters, shown in Figure 3c, are L_3_ = 9 mm, L_4_ = 9 mm, L_5_ = 38 mm, H_4_ = 7.85 mm, H_5_ = 12.8 mm, and G_2_ = 6 mm.

**Figure 4:**
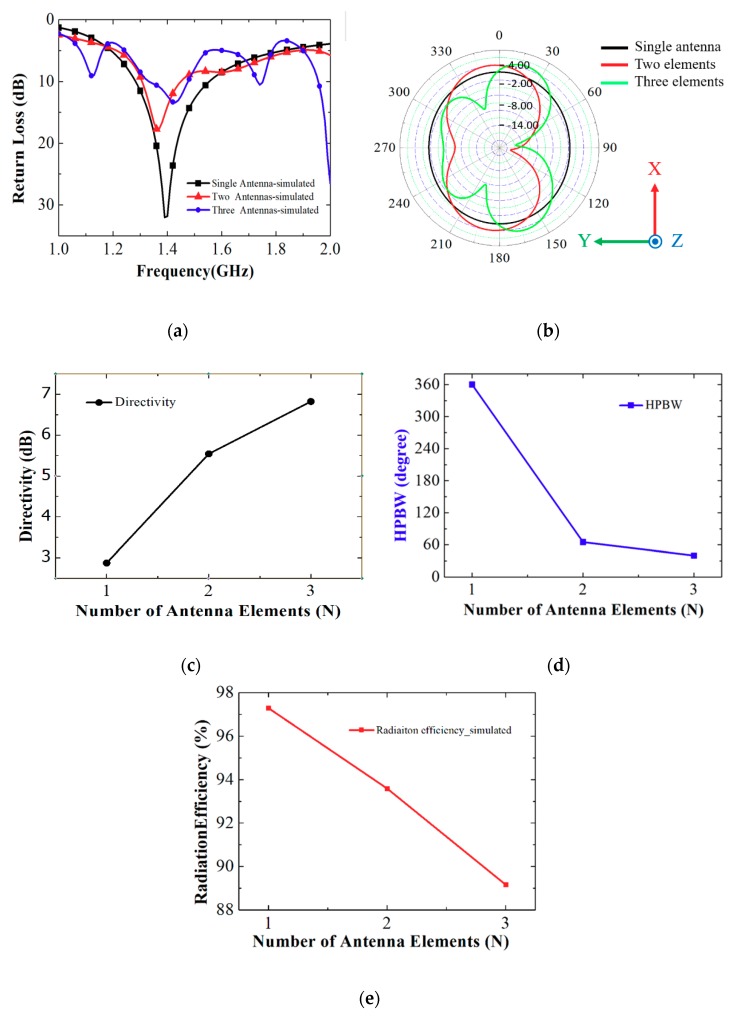
Simulated (**a**) return loss, (**b**) 2D radiation pattern on XY plane, (**c**) directivity, (**d**) half-power beam width (HPBW), and (**e**) radiation efficiency for different numbers of antenna elements of the antenna array.

Figure 4a shows the simulated return loss for the single antenna and for the two-element and three-element antenna array. The return loss was higher than 10 dB in the range of 1.29–1.54 GHz, 1.31–1.45 GHz, and 1.32–1.48 GHz for the single antenna and the two-element and three-element antenna array, respectively. Figure 4b shows the simulated 2D radiation patterns for the single antenna and for the two-element and three-element antenna array on the XY plane at 1.39 GHz; the peak gains were 2.91, 5.14, and 5.91 dBi, respectively; the half power beam widths (HPBWs) were 360°, 65°, and 40°, respectively. The asymmetric pattern of the three-element array was due to undesired radiation from the feeding lines. Figure 4c, d show the directivities and HPBWs for different numbers of the elements. Directivity increased and HPBW narrowed as the number of antenna elements increased, whereas the peak gain was only slightly increased above the three elements case due to the dielectric loss. The radiation efficiencies for the three loop antennas are presented in Figure 4e. The radiation efficiency decreased as the number of antenna elements increased because of the dielectric loss and feeding loss.

## 3. Fabrication and Measurement

**Figure 5:**
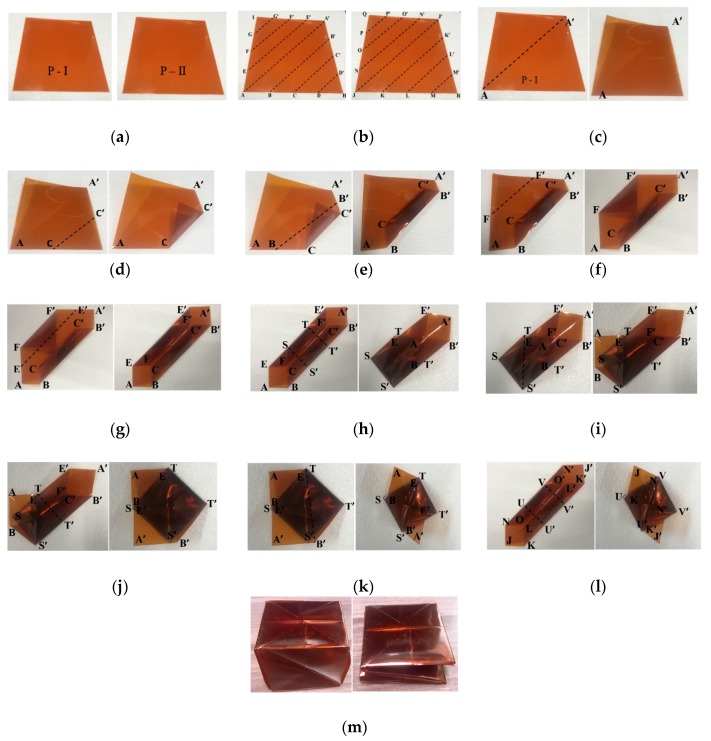
Magic cube origami folding process to fabricate the proposed single antenna element. (**a**) selection of two sheets of Kapton film, (**b**) sheets marked with alphabets, (**c**) folding sheet P-I along AA', (**d**) folding along CC', (**e**) folding along BB', (**f**) folding along FF’, (**g**) folding along EE', (**h**) dividing sheet into three segments and folding along S'S, (**i**) folding along TS', (**j**) folding along TT', (**k**) folding along TB', (**l**) folded sheet P-II, (**m**) unfolded and folded states for magic cube origami.

Figure 5 shows the origami folding process to fabricate the proposed single antenna element, as follows.

1. Two of the same 141 mm × 141 mm sheets of Kapton film, named P-I and P-II, were chosen as illustrated in Figure 5a.
  - Dividing Sheet P-I into sections AA', BB', CC', DD', EE', FF’, and GG’, as shown in Figure 5b-left. Subdivisions of P-II into JJ', KK', LL’, MM', NN', OO', and PP’ as shown in Figure 5b-right. P-Ⅰ sheet is folded along AA’ and unfolded across AA', it was divided into two right triangles, as shown in Figure 5c.
  - Folding along CC', then BB', FF', and EE', consecutively, produced hexagonal geometry with 4 shorter sides (AB, AE, A'B', A'E'), as shown in Figure 5d–g.
  - P-Ⅰ was separated into 3 segments comprising, as shown in Figure 5h.
    - Upper pentagonal section (A'B'T'TE'),
    - Middle square segment (TT'S'S),
    - Lower pentagonal section (ABS'SE)
    then folding along SS', the lower pentagonal section ABS'SE was folded in the direction of the middle square section TT'S'S, thus the sheet was converted into pentagonal geometry.
  - Folding EABS' along TS' in the direction of ESS', as shown in Figure 5i.
  - Folding section TE'A'B'T' downward along TT', as shown in Figure 5j.
  - Folding section TE'A'B' along TB' in the direction of TTB', as shown in Figure 5k.
2. Folding sheet P-II exactly as for P-I to create another hexagonal shaped folded geometry, as shown in Figure 5l. The origami magic cube in unfolded and folded states have been completed, as shown in Figure 5m.

**Figure 6:**
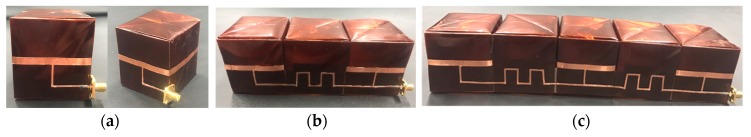
Fabricated samples: (**a**) Single antenna element, (**b**) two-element antenna array and (**c**) three-element antenna array.

**Figure 7:**
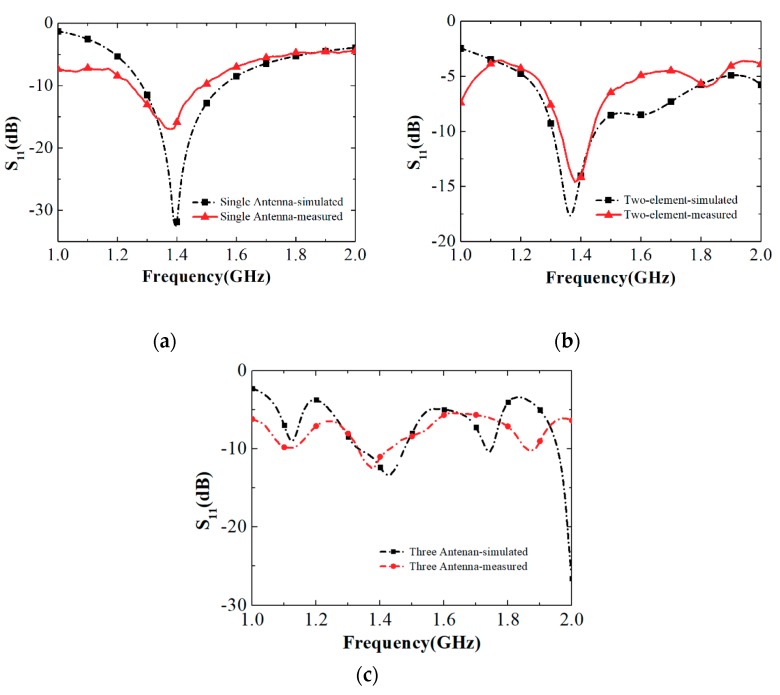
Simulated and measured S_11_ for (**a**) the single antenna, (**b**) two-element antenna array, and (**c**) three-element antenna array.

The complete antenna prototypes are exhibited in Figure 6. Figure 6a shows the side and perspective view of the single element antenna, whereas the 2-and 3-element antenna arrays are correspondingly shown in Figure 6b,c. The comparative simulation and measurement results of the reflection coefficient of the three antennas are indicated in Figure 7. In the unfolded state, the measured 10–dB impedance bandwidth of the single antenna was 18% (1.29–1.54 GHz) showing a good agreement with the simulation result of and 17% (1.25–1.48 GHz). As can be seen in Figure 7b, the measured 10-dB impedance bandwidth of the 2-element array was 10% (1.31–1.45 GHz), whereas it was 5% (1.34–1.43 GHz) obtained in the simulation. As the number of elements increased, the difference between the simulation and measurement results increased because of the assembly error. This problem can be solved by using thinner semi-flexible materials.

**Figure 8:**
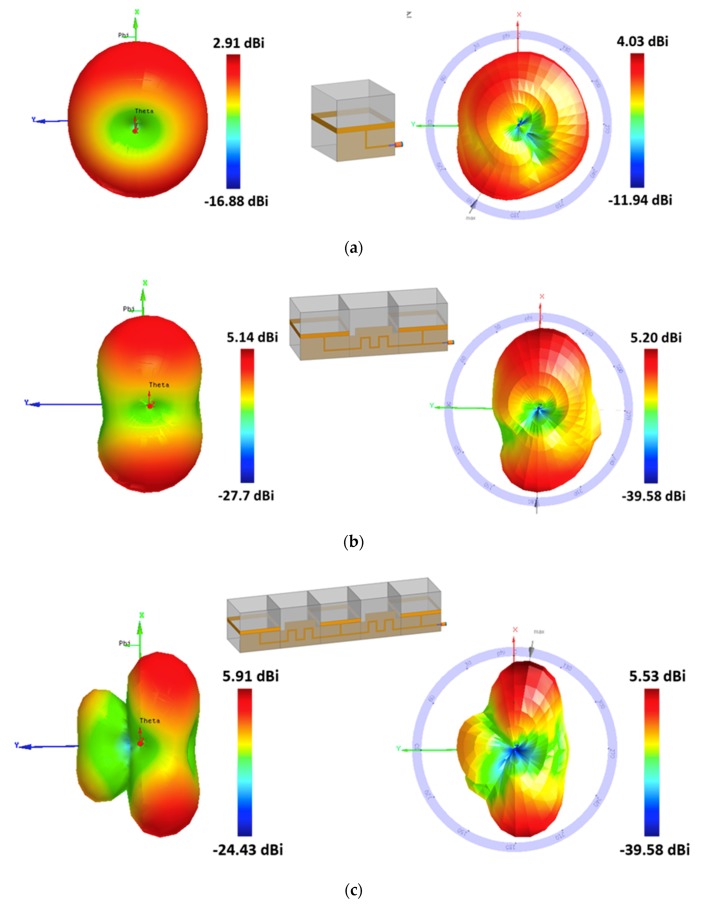
Simulated and measured 3D radiation patterns at 1.39 GHz for (**a**) the single antenna and (**b**) two-element antenna array and (**c**) three-element antenna array.

Figure 8 shows the simulated and measured 3D radiation patterns plotted at 1.39 GHz. The measured peak gain for the single, 2-element, and 3-element were 4.03 dBi, 5.20 dBi, and 5.53 dBi, respectively, and the measured HPBW were 360°, 65°, and 40°, respectively, i.e., the peak gain increased, and the beam shape became sharper with an increasing number of antenna elements. The overall thickness for the 3-element array was 250 and 25 mm for the unfolded and folded states, respectively. Thus, the proposed antenna array can be folded compactly for deployment and unfolded to provide full mode when used.

## 4. Conclusions

This paper proposed a deployable high gain loop antenna array using magic cube origami, producing a bi-directional radiation pattern. To increase directivity, the antenna array was comprised of cascading magic cubes. Since each antenna element was realized on the single magic cube, we designed a serially fed antenna array of connected magic cubes. The single-, two-element antenna arrays were fabricated by the magic cube origami folding process. The peak gain of the single-, two-, and three-element antenna arrays were4.03 dBi, 5.20 dBi, and 5.53 dBi, respectively. In addition, the measured HPBW of the single-, two-, and three-element array were 360°, 65°, and 40°, respectively. The magic cube geometry allowed the proposed antenna array to be easily folded to minimize the overall volume. The overall thickness of the three-element array was 250 mm and 25 mm for the unfolded and folded case, respectively. Therefore, the proposed antenna structure provides significant advantages for easy transport and deployment. It is successfully demonstrated that the peak gain is increased and HPBW is sharpened with a higher number of antenna elements up to the three-element antenna array. However, the high sidelobe and asymmetric pattern are observed from the radiation pattern of the three-element array because of the parasitic radiation from the feeding lines. Therefore, the proposed idea is working up to the two-element array in terms of directivity, peak gain, HPBW, and radiation patterns.
